# Informing Culturally Safe Advance Care Planning: An Interpretive Descriptive Study of Internationally Educated Nurses in Ontario

**DOI:** 10.1177/08445621241278922

**Published:** 2024-09-12

**Authors:** Shereen Jonathan, Kathryn Pfaff, Edward Cruz

**Affiliations:** 1 Faculty of Nursing, University of Windsor, Windsor, ON, Canada

**Keywords:** Cultural safety, advance care planning, internationally educated nurses, end-of-life planning, cultural humility, empowerment

## Abstract

**Background:**

Maintaining cultural safety during advance care planning (ACP) discussions is an essential component of holistic care provision. Most nurses feel unprepared to engage in ACP and the current literature offers limited recommendations on how nurses can lead culturally safe ACP discussions. Internationally educated nurses (IENs) have unique personal and professional experiences to address this gap.

**Purpose:**

The purpose of this study was to understand IENs’ contributions to culturally safe ACP and its implications to nursing practice and ACP policy.

**Methods:**

An interpretive descriptive approach was undertaken. Ten IENs working in Ontario, Canada were individually interviewed using a semi-structured guide to understand their perspectives and experiences of engagement in culturally safe ACP practices.

**Results:**

IENs utilized various approaches that were reflected in three actions: practicing cultural humility, utilizing a cautious approach, and empowering clients and families. IENs engaged in intrapersonal and interpersonal cultural humility practices to recognize the unique influence of one's culture on the ACP process. Establishing trust in the nurse-client relationship and cautiously approaching ACP conversations was recognized as important in maintaining cultural safety. IENs also empowered clients by addressing knowledge deficits, misconceptions about ACP, and informing them of their decision-making rights.

**Conclusion:**

Nurses require education and resources to carry out culturally safe ACP. Education should begin at the undergraduate level and include self-engagement in ACP and cultural humility training. Practicing nurses need ACP training and clear standards/guidelines. There is an opportunity for healthcare organizations and professional/governing nursing bodies to collaborate on developing culturally safe ACP guidelines.

## Background and purpose

Planning for one's end-of-life can often be a complex and sensitive topic among the general population. Early end-of-life (EOL) planning, also known as advance care planning (ACP), enables one's values and future care wishes to be upheld during the last phases of life (Canadian Hospice Palliative Care Association, 2015). ACP is defined as “a process that supports adults at any age or stage of health in understanding and sharing their personal values, life goals, and preferences regarding future medical care” (Sudore et al., 2017, p.826) to facilitate care that align with their goals, values, and preferences (Sudore et al., 2017).

Culture has been reported to have a more significant influence on the ACP process than factors such as age, education, and socioeconomic status (Nayfeh et al., 2019). Facilitating ACP discussions that respect a person's culture is an essential component of culturally safe client-centered care, and these discussions can benefit families, healthcare professionals, and the healthcare system (Nayfeh et al., 2019). The term cultural safety was first introduced by Dr. Irihapeti Ramsden in the 1990s as delivering “quality care through changes in thinking about power relationships and patients’ rights” (Curtis et al., 2019). Cultural safety aims to preserve one's cultural values and beliefs and is “based on respectful engagement that recognizes and strives to address power imbalances…[and] where people feel safe when receiving health care” (First Nations Health Authority, n.d.).

Cultural humility is highlighted in the literature as an essential aspect of achieving cultural safety. Cultural humility challenges the practitioner to reflect on their clients’ as well as their own health beliefs and values along with its influence on care expectation and provision (So et al., 2024). Engaging in cultural humility fosters an environment that prioritizes safety and inclusivity in healthcare (So et al., 2024).

A systematic review by Hong and colleagues (2018) established that culturally diverse groups reported poor accessibility, awareness, and knowledge about ACP. Various minority groups also expressed the view that ACP would not enhance quality of life and facilitate care that aligns with their wishes (Hong et al., 2018). In looking for avenues to address these barriers and carry out ACP in a culturally safe manner, current literature lacks clear recommendations on how culturally safe ACP should be undertaken by nurses. They largely offer vague recommendations such as respecting an individual's cultural values and beliefs about medical care and carrying out approaches based on an individual's values (Nayfeh et al., 2019). Recommendations offered in the literature are mostly educational in nature, generalized to all healthcare providers, and lack practice-based interventions (Fang et al., 2016). Failing to uphold cultural safety can lead to distrust in the healthcare system and negatively impact a person's healthcare experience (Richardson & Williams, 2007).

The literature suggests that nurses play an important role in engaging clients and families in ACP (Fliedner et al., 2021; Izumi, 2017). Nurses identify that initiating and facilitating ACP discussions is within their scope of practice (Fan & Rhee, 2017; Rietze et al., 2018). The Hospice and Palliative Nurses Association (2011) recognize nurses’ ethical responsibility to advocate for client's EOL wishes to promote optimal health outcomes. Canadian professional and regulatory organizations also outline general practice competencies that include identifying a person's needs and beliefs about health to inform holistic client-centered care plans across the lifespan (Canadian Association of Schools of Nursing, 2011; College of Nurses of Ontario, 2019a, 2019b, 2019c).

Internationally educated nurses (IENs) are an important source for informing culturally safe ACP practices. IENs are generally recognized as nurses who have completed their initial nursing education in a country other than where they are currently practising. IEN contributions to research have been used to inform nursing practice and enhance the delivery of care to ethnically diverse individuals. Findings from a descriptive phenomenology qualitative study conducted by Njie-Mokonya (2016) found that IENs demonstrated cross-cultural competence and their shared experiences informed and enhanced understanding on cross-cultural practices and care provision. IENs are in a unique position to speak on ACP engagement with clients who maintain varying cultural values and beliefs. IENs hold various cultural beliefs and practices themselves and have had experience providing care in multiple countries to ethnically and culturally diverse individuals. Giving IENs an opportunity to speak about their ACP experiences with clients is a step towards designing cross-cultural nursing approaches to facilitate culturally safe ACP and addressing existing barriers of poor ACP engagement among culturally diverse individuals.

Therefore, the purpose of this study was to understand IENs’ contributions to culturally safe ACP and its implications to nursing practice and ACP policy. This study answered the following three research questions: (1) What are IEN's experiences of engaging in ACP in their home country and how do they compare with their experiences in Ontario? (2) What culturally safe ACP practices could help preserve and maintain individual/family cultural values and beliefs? (3) How can ACP practices be re-structured to support culturally safe ACP in Ontario?

## Methods and procedures

### Design

This study utilized an interpretive descriptive approach (Thorne, 2016). This noncategorical qualitative research approach goes beyond description and aims to apply the knowledge attained towards informing practice (Thorne et al., 1997). This supports the production of evidence that is pertinent and applicable to current nursing practice.

### Sample

IEN participants were recruited through convenience and snowball sampling methods. Participants had completed their initial nursing education outside of Canada and the United States and were currently practicing as either a registered nurse (RN) or registered practical nurse (RPN) in Ontario, Canada. In Ontario, RPNs are expected to complete a two-year diploma-based program and RNs are either diploma or baccalaureate prepared (Registered Nurses’ Association of Ontario [RNAO], 2024). RNs who have graduated after 2005 have completed a four-year baccalaureate degree (RNAO, 2024). RNs have a more comprehensive knowledge base compared to RPNs, and are better equipped to care for clients with more complex and critical heath care needs (RNAO, 2024).

Participants had been working in Canada as a RN or RPN for at least three years and reported having experience engaging in ACP in their practice. Participants were recruited by email and a recruitment flyer through two community colleges located in Central East and Western Ontario that offer bridging programs for IENs, the CARE Centre for IENs, and a private Canadian IEN Facebook group. The IEN sample consisted of individuals from various cultural backgrounds who had completed their primary nursing education in different countries. Final sample size and data saturation was determined based on data redundancy and when the research team determined that the research questions were answered (Thorne, 2016). The final sample consisted of 10 IENs.

### Data collection

Data collection occurred during the COVID-19 pandemic from August 2020 to November 2020. In-depth semi-structured phone interviews were completed by the primary author at her home privately and consisted of open-ended questions and related probe questions. These questions were designed by the team to capture IENs’ ACP experiences and were based on the concepts outlined in Leininger's sunrise model (Leininger, 1997).

The sunrise model supports a culture care worldview that can be influenced by various cultural and social structural dimensions (Leininger, 1997). Leininger states that these dimensions influence care expressions, patterns, and practices within cultures, which then influence one's perception of their holistic health and overall well-being (Leininger, 1997; Leininger & McFarland, 2006). These concepts ultimately contribute to one's cultural care worldview and impacts the delivery of nursing care (Leininger, 1997; Leininger & McFarland, 2006). This model also explores various aspects of nursing care including generic care- familiar/beneficial practices learned over time and professional care- practices learned through the professional education system (Leininger & McFarland, 2006). These learned practices contribute to three nursing decisions and actions that aim to preserve, negotiate, and restructure an individual's cultural care provision needs and wishes. The goal of this model is to provide culturally congruent care that will contribute to one's health and well-being (Leininger, 1997; Leininger & McFarland, 2006). Utilization of these concepts in Leininger's sunrise model were undertaken in this study to learn about IENs’ approaches towards culturally safe ACP. Questions inquiring about ACP knowledge and work background/experiences were also included. See Appendix A for interview question guide.

After the initial two interviews, questions were reviewed again by the team and additional probe questions were added to help enrich data collection. Member checking was not carried out in this study; rather emerging conceptualization of themes, instead of raw data, were presented to some participants to receive constructive feedback (Thorne et al., 1997). Interviews lasted approximately 20 min to one hour and took place in one sitting. After receiving informed verbal consent, interviews were audio recorded on a device used solely for this study, and notes were taken during each interview to document emerging ideas. Participants were sent a $20 Amazon e-gift card after the interview as a token of appreciation.

### Data analysis

Analysis involved an inductive and iterative approach (Thorne et al., 1997) of reviewing the transcripts, along with coding and querying data using ATLAS.ti 9 software. Each transcribed interview was reviewed prior to coding, during analysis, and when identifying final themes. Initial coding was completed by the primary author and subsequent feedback was received by the co-authors on these initial and emerging codes. The iterative data analysis process involved concurrent immersion in data collection and data analysis as well as active challenging of emerging conceptualizations (Thorne et al., 1997, 2004). The use of small units of analysis and premature coding were avoided (Thorne et al., 1997). Refer to coding tree in Table 1.

**Table 1.** Coding tree.

Examples of Initial Codes	Categories	Final Themes
Understanding of one's own cultural values and beliefs Recognition of one's own biases Understanding that cultural values and beliefs differ from person to person Asking questions about clients’ cultural values, beliefs and practices Non-judgemental attitude Noting non-verbal cues	Self-reflection Awareness of differing cultural views Seeking to learn about clients’ and families’ cultural values, beliefs and practices	Cultural Humility
Establishing and maintaining trust with clients Initiating ACP conversations with general care discussion topics Taking the extra time to have ACP discussions Slowly carrying out ACP discussions Assessing interest in ACP Ensuring clients feel respected during ACP discussions	Establishing a professional nurse-client relationship Carefully approaching ACP	Being Cautious
Involving members of the healthcare team Explaining the process and importance of ACP engagement Addressing knowledge deficits and misconceptions related to ACP Providing ACP resources	Advocating for clients and families Informing clients of their rights to informed decision-making and appointing a substitute-decision maker	Empowerment

### Reflexivity and ethics

This study contributed to the primary author's Master of Science in Nursing thesis. Employed as a Baccalaureate-prepared RN, the primary author designed the study in direct consultation with and under the training of her co-advisors Dr. Kathryn Pfaff and Dr. Edward Cruz. The primary author had no prior relationship with any of the participants and no details about the primary author's personal life or interests in the study were disclosed to participants. This study received ethics clearance from The University of Windsor and the Multi-College Research Ethics Board. Bracketing was undertaken to mitigate the influence of researcher bias and preconceptions during data collection and analysis. A reflective journal (Thorne et al., 1997) was kept throughout data collection and analysis to document decision points, progress, and self-awareness of the potential influence of previous knowledge and experiences on the findings.

## Results

IENs sampled in this study included three RPNs and seven RNs. At the time of the study, the highest education level held by participants were RPN diploma (n = 3), Bachelor of Science in Nursing (n = 6) and Master of Nursing (n = 1). These participants received their initial nursing education from different continents including Asia (n = 6), Africa (n = 2), and Europe (n = 2) and were employed as nurses anywhere from seven to 40 years. Specifically, participants were employed in the country where they received their initial nursing education anywhere from two to 22 years and three to 32 years in Canada. Nine out of 10 participants had acute care experience. Two individuals, not included in the sample, initially contacted the researcher to participate in this study but were later unable to participate due to personal time constraints.

Firstly, IENs shared clinical experiences of engaging in ACP with individuals in their home country and outside their home country. Next, three main themes were identified as culturally safe ACP practices by IENs: fostering cultural humility, being cautious, and empowering clients. Lastly, IEN thoughts on how nurses can be supported in engaging in culturally safe ACP will also be discussed.

### IEN experiences

IENs in this study consistently expressed that ACP was non-existent or poorly carried out in their home countries. Decision-making regarding care provision, especially at the time of diagnosis, change in health status, and at EOL was usually family-centered. Autonomous decision-making was not valued nor exercised in their home countries. Based on the wishes of the family, individuals often never learned of their critical illness/condition diagnoses. In most instances, designated family member(s), chosen as a result of cultural norms and tradition, oversaw health care decisions for their loved one. Participant one, an RPN with 12 years of experience in acute care and long-term care settings, expanded on the poor ACP engagement in their home countries:

“Advance care planning back home is non-existent because our culture believes that when somebody would have a certain illness, the family members would take care of them. They don’t have decent values of independence and quality of life…. at that point, they will just, the person having diagnosed with a certain illness, they just slip into that dependent role to their caregiver. I should say that advance care planning is very not existent in our culture.”

Participant five, a RN with 24 years of experience in acute care, shared the following:

“…in South Africa, it's always the gogo like your grandmother, you don’t get to choose. It's tradition, it is what it is…. Yeah, it's not that I choose the person, it's just who it is, that's how the system works there.”

Participant eight, an RN with 13 years of clinical experience in acute care, added:

“In a family, there is always somebody that you respect and not necessary to be written, usually the oldest man in the family, but if there is no man, then it would be the oldest woman. Still the choice cannot be with one person, they will share with all family members.”

Three participants suggested that the reason engaging in ACP was not a valued practice in their home countries may be due to the fact that the standard of care expected was to ensure that all measures of care are to be carried out, regardless of the client situation or prognosis.

“Even deciding care, it's usually what everyone wants- to be like resuscitated or do the maximum to like save their life even if they are really old or something, even if they are sick and if they don’t have a chance to survive, they prefer full resuscitation” (P3).

Participant eight echoed this and inferred that the act of ensuring that all measures of care were being carried out for a loved one was important in expressing their love and affection.

“The affection and the emotional relationship…. doing things, going through all approaches, even seeking even national help, meaning like some people I know won’t stop with one doctor or two, they go all over the country or even outside the country for more help before they agree to just accept end-of-life and accept the local treatment by comfort measure. Because they want to extend life as much as possible. That's how they believe, it is their affection expression.”

Reflecting on their experiences engaging in ACP with clients in Canada, IENs found similar barriers such as lack of ACP awareness and opposition to be present.

“People still have their same values in their culture with them. It's the type of values of the family, that is the big challenge because if they are not open to it and are not aware of what advance care planning is about then that is a huge barrier.” (P1).

“When it came to specific cultures, I found that a lot of people were not very open to having that conversation because they never saw end of life as something that-they were not comfortable about end of life. They knew that someday they were going to die for sure, but they never talked about it, because it was taboo, something that you don’t talk about even if it's going to happen for sure. Even in my culture, people don’t like talking about it.” (P6).

However, in Canada, IENs explained that ACP was more holistic and supported by the healthcare team in carrying out client's wishes. IENs also expressed that they were better equipped and felt more comfortable having ACP conversations in their practice in Canada. Participant six, an RN with 19 years of experience in acute care, long-term care, and community settings, shared thoughts on this:

“I would say that when I started working in Canada…it was a lot different and well accessible. I felt less restricted in discussing ACP with my patients, I also had more support from my team to carry out their wishes.”

Participant three, an RPN with 14 years of experience in acute care, stated:

“I think there is more opportunity in here to have ACP discussions with people from different cultures, especially with people who are hesitant to discuss ACP and it is not usual in their culture to discuss this. Me being from a different ethnicity and culture and my patients seeing that, they are more willing to talk about ACP and I also share why ACP would be good for them.”

IENs drew from their home country experiences of facing strong opposition towards ACP engagement and adapted their approaches to uphold cultural safety in ACP conversations within their current practice.

### Fostering cultural humility

While acknowledging the influence of culture on the ACP process, IENs shared various practices to mitigate potential barriers and maintain cultural safety during ACP discussions. Five participants described a variety of intrapersonal and interpersonal cultural humility practices in support of ACP engagement with their clients. Approaching ACP conversations from a stance of cultural humility allowed IENs to acknowledge their own unique cultural values and practices along with preconceived notions and biases regarding other individuals’ cultural standpoint and practices. In this manner, IENs also placed importance on developing an understanding of clients’ cultural worldview.

Intrapersonal cultural humility practices included self-reflection and a heightened awareness that another individual's cultural views could differ from their own.

“You have to make sure you don’t project your own perspective. That is why I believe every nurse should reflect on their own personal values in their culture because sometime you are unaware that it is affecting your nursing care.” (P1).

Participant six, an RN with 19 years of experience in acute care, long-term care, and community settings stated:

“I do a lot of self-reflective practice in the sense that I know that I have a culture, I come from a culture and with my own bias. So I am not going to judge someone when going to have this conversation because I know I have a practice because of my culture. So I have that expectation that the person I am having the conversation with, would definitely already have a cultural practice, which would affect advance care planning.”

IENs acknowledged that each individual's cultural worldview was unique and needed to be identified and valued during the ACP process. Interpersonal cultural humility practices involved intentional learning of one's unique cultural values and beliefs to avoid making assumptions and generalizations.

“…some cultural humility is always a good thing, being ready to hear what someone has to say rather than telling them what you want them to do… I would explore what they wanted rather than assuming I knew what they wanted.” (P7).

“It would be better for people to understand advance care planning from their patient's cultural perspective. Every culture is different, so that's where we should be looking at. I don’t think we should impose advance care planning conversations on people without considering an individual's cultural background or beliefs.” (P6).

“I have changed my focus to try and understand what kind of cultural practices and beliefs the patient has and that's how I start the [ACP] conversation. I do this so I don’t by mistake generalize or guess what their beliefs might be based on their ethnicity.” (P3).

An approach of cultural humility relayed to clients and families that their cultural beliefs are recognized, valued, and facilitated continued ACP discussions.

### Being cautious

Another practice to maintain cultural safety involved being cautious during ACP discussions. In preparation to initiate ACP conversations, three IEN participants found it important to build a professional nurse-client relationship which was supported by adopting a stance of cultural humility previously discussed. This was important to foster trust and form a connection where clients and families feel comfortable discussing ACP topics. Participant five shared the following:

“I don’t just start talking about advance care planning or end of life, it starts from the one-on-one nurse patient relationship, right? When the patient is comfortable with me, that's when I start engaging them in more conversations about advance care planning… I first find out if the person is interested in having an advance care planning conversation and then I go from there.”

Once a professional relationship was established, five participants discussed using a cautious approach. Participant one shared how they approached ACP conversations with ethnically diverse individuals:

“I have to be slow and explain everything, try to gain their understanding, try to acknowledge their questions because at that point I will be able to discuss or elaborate further or make a shift in their perspective.”

This cautious approach of carrying out ACP discussions allowed IENs to first grasp a clear understanding of individuals’ and families’ understanding of ACP and then take the opportunity to further discuss ACP as appropriate. IENs also discussed this was significant especially when nurses are trying to understand one's position on ACP and to assess their willingness to engage in ACP conversations. Participant eight explains:

“I will take the time to really see their [ACP] understanding and take a look at different approaches before I propose it. This is to respect their feelings… [it] makes my interaction with people from different religion or different culture really easy or much easier to manage.”

Participant seven, an RN with 40 years of experience in acute care and community settings, expanded on this approach:

“I get a feel for what their [care] expectations are, what their trajectory might be and then, I might ask them about things that they want to do or what's important to them at this point… what is it you want to see? Have you thought about that kind of thing?”

Approaching conversations in this cautious manner allowed IENs to initiate ACP discussions at an appropriate time and tailor ACP discussions based on clients’ and families’ knowledge and understanding of ACP. This led to greater success in working towards an ACP that met their needs and care expectations.

### Empowering clients

To address health inequities, participants discussed their efforts to empower individuals and recognized this as an essential component in facilitating culturally safe ACP. Various ways to empower clients were discussed by four participants which involved notifying clients of their rights to informed decision-making and appointing a substitute decision-maker, expressing the importance of ACP, and addressing knowledge deficits. Participant Six discussed the significance of ensuring that clients are informed of their right to voice their wishes and be involved in making decisions pertaining to their own health and well-being:

“…we want to be able to empower the individual to know that [they] have a right of knowing that the person who is going to make decisions for [them] would be making that decision in [their] best interest. That's how we can incorporate culture in having that conversation. We do respect [their] culture… how would [THEY] feel, what do [THEY] want as a person, what would [THEY] like to see happen. We want to empower each person while respecting their culture, and I think this is a way to do it.”

IEN participants also sought to empower clients by conveying the importance and benefits of ACP engagement and providing resources which are available to them in their workplace or online to help address knowledge deficits or misconceptions that clients may have about ACP.

“I also find that I have to explain the difference between power of attorney for finances and power of attorney for personal care and terms like that. So there can be confusion about the legal standing of a person making decisions on your behalf. So you sometimes have to go into that a bit and I do refer them to the government website because there is a booklet. And we have paper copies as well if people want them… then I explain why it's important that they talk about [ACP]…People mostly don’t realize what advance care planning is and have the wrong idea about it” (P7).

Involving members of the healthcare team in the ACP process were also discussed by four participants. IENs collaborated with physicians, social workers, physiotherapists, occupational therapists, pharmacists, dietary, and chaplains in the ACP process. These healthcare team members were involved for their areas of expertise in helping address the needs and wishes of clients and families and share practices that they had found to be effective in carrying out culturally safe ACP.

### Restructuring culturally safe ACP: engagement, education, and Resources

IEN participants had the opportunity to share strategies to support nurses in carrying out culturally safe ACP with their clients. Personal engagement in ACP was mentioned to be an essential practice for understanding and maintaining a culturally safe ACP process. Participant Seven explained:

“…I think it would be a good idea for the person who is asking others to engage in these conversations to have done this for their own family and their own decisions… because, until you do it, you don’t understand how facing these questions makes you feel.”

Six IEN participants recognized that new graduate nurses struggle initiating and carrying out ACP with clients and families. To address this, IENs talked about the need for undergraduate nursing students to build their knowledge and practice engaging in ACP conversations as part of baccalaureate and practical nursing curriculums.

Within current practice, IENs spoke about the need for incorporating similar culturally safe ACP education into nurses’ continuing education credit requirements in the form of workshops, seminars, or conferences. In addition, three participants also stated the need for best practice guidelines to establish their role in the ACP process and provide recommendations on how nurses should carry out culturally safe ACP.

“If there were something like that so nurses could refer to… I think that best practice guidelines would be really helpful, that way you are all on the same page trying to get to the same point. Rather than everyone doing what they think is the best thing.” (P7).

Out of the ten participants in this study, only three participants identified having ACP resources in their workplace, either in the form of hardcopy materials or resources readily available to the public online. These resources available were recognized to be generic and lacking cultural perspectives that need to be taken into consideration during the ACP process. Seven participants stated that they did not utilize any ACP resources, nor were they made available to them in their workplace.

## Discussion

The findings from this study illuminate IEN perspectives on carrying out culturally safe ACP with clients and families in their practice. New practical findings have emerged that contribute to the growing body of knowledge on culturally safe ACP.

IENs in this study painted a stark comparison of ACP experiences with ethnically diverse individuals in their home countries versus their experiences in Canada. To our knowledge, this is the first study to compare IENs’ unique experiences of engaging in culturally safe ACP in different countries and how it has influenced their current practice. IENs iterated that in their home countries, ACP was not viewed as a beneficial process in which to engage. Planning for EOL was rarely discussed and regarded as an extremely sensitive topic that was rarely broached upon. Client autonomy was poor and individuals were rarely involved in decision-making pertaining to their care and opted to have an appointed family member(s) make care decisions on their behalf. The standard and expectation of care was that all medical interventions were to be carried out for clients regardless of their prognosis or future quality of life. Overall, clients and families displayed a great lack of awareness and knowledge about ACP including its benefits to tailoring care provision to proficiently meet their unique needs and wishes.

In Canadian practice, IENs faced similar ACP engagement barriers such as lack of awareness regarding the ACP process and benefits of ACP engagement, as well as reluctance towards speaking about EOL topics. Comparatively to home country experiences, IENs had more positive experiences engaging in culturally safe ACP. IENs expressed having greater support from the healthcare team to work cohesively to meet the needs and wishes of clients. IENs felt more comfortable engaging in ACP as they drew on their previous home country practice experiences which included facing clients’ and families’ barriers and oppositions to ACP. This allowed them to focus on carrying out ACP discussions in Canadian practice with a greater awareness on the influence of culture and approach ACP in a manner which encompassed culturally humility, cautiousness, and empowerment.

Engagement in cultural humility practices and its impact on culturally safe care in general is a finding that has been acknowledged in the literature (Chang et al., 2012; Singh et al., 2023). However, this study provided further insight on certain cultural humility practices IENs valued in the unique process of ACP. Cultural humility is “a life long process of openness, self-awareness, being egoless, and incorporating self-reflection and critique after willingly interacting with diverse individuals” (Foronda et al., 2016, p. 213). These authors state that results of achieving cultural humility include: mutual empowerment, partnerships, respect, optimal care, and lifelong learning. IEN participants engaged in various intrapersonal and interpersonal cultural humility practices to facilitate ACP engagement.

IENs discussed self-reflecting on their own culture and how associated personal biases and practices could impact carrying out ACP discussions with clients. Engaging in intrapersonal cultural humility practices such as self-reflection of biases, assumptions, and stereotypes has been shown to be important in the path towards culturally humility and essential in addressing power and health inequities (Chang et al., 2012; Singh et al., 2023). IENs also shared that self-reflection of past ACP experiences from their home country, specifically the challenges and barriers they faced during ACP engagement, impacted their current approach of ACP with culturally diverse individuals.

Regarding interpersonal cultural humility, IEN participants sought to understand each client's culture and associated beliefs/practices. This was recognized to be important in maintaining cultural safety in the ACP process, which has also been discussed in the literature (Chang et al., 2012, Nayfeh et al., 2019; Singh et al., 2023). Nayfeh and colleagues (2019) report that understanding each other's belief system was an effective cross-cultural strategy for building trust when engaging in ACP discussions regarding mechanical ventilation. Although IEN participants valued learning about individuals’ culture and associated needs, values, and EOL care expectations, participants did not place any importance on sharing their own cultural values and beliefs with their clients. A few participants additionally expressed that in their past experiences, sharing personal cultural values and beliefs with clients often negatively impacted ACP discussions. This was due to crossing professional boundaries leading to increased hesitancy and unwillingness on clients’ behalf to engage in ACP discussions.

While the general concept of cultural humility has been discussed previously in the literature in maintaining cultural safety, IEN participants specifically discussed that engaging in cultural humility practices were important in strengthening professional nurse-client relationships to foster trust with clients. Building a strong nurse-client relationship as a product of practicing cultural humility helped set the stage for IENs to approach ACP discussions in a culturally safe manner.

Another approach discussed by IENs in this study was upholding cautiousness in the timing and overall discussion of how ACP discussions were carried out. A systematic review by McDermott and Selman (2018) exploring the cultural influence on ACP and how ACP could be approached cross-culturally suggested using a broad communication approach as opposed to a process focusing on formal documentation. Although IEN participants sought to understand clients’ cultural values and beliefs as part of culturally safe ACP, they did not use a broad open approach. IENs recognized the sensitivity required when discussing EOL planning and expressed the importance of an approach that exercised cautiousness during ACP conversations. Being cautious specifically involved recognizing potential barriers of ACP engagement and cultural norms that influence ACP engagement and ascertaining clients’ position and willingness to engage in ACP. This approach was deemed necessary by IENs based on their foreknowledge and past experiences of consistent opposition when approaching ACP discussions with culturally diverse individuals in their home countries.

IEN participants also emphasized the significance of empowering clients and families throughout the ACP process. Empowering clients through a focus on care delivery by changes in thinking about power relationships and client rights is an essential aspect of cultural safety (Curtis et al., 2019). The ACP process has been recognized as a means to empower clients and families (Goswami, 2023); however, the literature lacks recommendations on how nurses can practically empower individuals during the ACP process.

IENs in this study elaborated on how they sought to empower clients and families during ACP. IENs expressed that common barriers to ACP among culturally diverse individuals was a lack of general knowledge regarding ACP and misunderstanding that ACP would not respect their cultural values and beliefs. Therefore, when seeking to empower clients, IENs sought to clarify these misconceptions and address ACP knowledge deficits. This involved providing education on power of attorney and other related legal terminology as well as helping clients and families develop an understanding of the purpose and general process of ACP. IENs also emphasized the value of ACP engagement to clients in receiving care that aligns and respects their cultural values and beliefs. Supporting clients in appointing substitute decision maker(s) and encouraging involvement in informed decision-making were also viewed as important when empowering clients.

In this study, collaboration within the healthcare team also facilitated client empowerment and allowed nurses to better engage in ACP discussions through the sharing of practical strategies that can used to maintain cultural safety. Interprofessional collaboration was also recognized by IENs as essential to meeting clients’ holistic needs that fell outside their scope of practice. This is an important finding as interprofessional collaboration in ACP is currently limited to its value in engaging ethnoculturally diverse team members as interpreters when language barriers with clients/families are present (Nayfeh et al., 2019).

### Implications

IENs called for greater resources and supports for nurses in carrying out culturally safe ACP and voiced the need for more general ACP education and cultural humility training in their practice.

The rainbow model of cultural humility developed by Foronda (2020) outlines a guide for nurses to achieve cultural humility in their practice. This model establishes diversity as its worldview and explores contexts such as historical precedent, political climate, personal beliefs, physical environment, and situation. These contexts differ among individuals and communities, leading to power imbalances and cultural conflict (Foronda, 2020). Applying the action and decision of cultural humility leads to positive outcomes such as mutual empowerment, partnerships, respect, optimal care, and lifelong learning (Foronda, 2020). This model, representing the essential aspects leading to achieving cultural humility, should be included in undergraduate nursing education and IEN bridging curricula. This would enable undergraduate nursing students and IENs transitioning to Canadian practice to understand the factors leading to power imbalances and cultural conflict as well as the positive impact of adopting a stance of cultural humility.

In addition to addressing knowledge deficits surrounding cultural humility, nurses must engage in various practices to foster cultural humility. At the undergraduate level, nursing students should be provided with opportunities to develop cultural humility practices to utilize in their profession. Reflective journaling enables greater awareness among undergraduate students of the influence of culture on care planning and a deeper understanding of the impact of health disparities on health and well-being (Schuessler et al., 2012). Global service training has also been shown to support undergraduate nursing student engagement in cultural humility practices (Matthew et al., 2018; Sedgwick & Atthill, 2020). Since achieving cultural humility is a life long process, incorporating cultural humility training and reflection into workplace training and continuing education should be strongly considered.

Our findings suggest that self-engagement in ACP is important to develop a deeper understanding of the ACP experience and better support individuals and families in practice. As the literature demonstrates a significant correlation between nurses’ ACP knowledge and engagement of ACP with clients (Shepherd et al., 2018), nurses must also be provided with opportunities to address general ACP knowledge deficits. A systematic review of the literature suggests that interventions such as discussion-based education, online tutorials, e-simulations, and role playing can improve ACP knowledge, communication skills, comfort, and confidence in engaging in ACP discussions among healthcare providers (Chan et al., 2019). Competency-based ACP training including these interventions should be considered at the undergraduate and professional level.

With regard to policy, best practices/standards on carrying out culturally safe ACP are lacking and needed. Nurses are often unclear about their role in the ACP process (Beck et al., 2017; Fan & Rhee, 2017), how to go about carrying out ACP in their practice, and are concerned about a lack of guidelines and institutional policies (Fan & Rhee, 2017; Rietze & Stajudhar, 2015; Zhou et al., 2010). Existing guidelines are often generic and fail to meet the cultural needs of individuals and families (Nayfeh et al., 2019). Greater acknowledgment of the influence of culture on ACP and incorporating culturally safe guidelines and practices must be initiated by relevant organizations as well as regulatory and professional nursing bodies. IENs in this study stated that ACP materials to support culturally safe ACP are greatly lacking and if available are focused on completing legal advance directive documentation and fail to capture the holistic nature of ACP. There is a current need for ACP materials to support the delivery of culturally safe ACP in clinical practice.

### Limitations

This study utilized a small sample size; therefore, transferability of findings is limited. Since English was not a first language for many participants, fully grasping participants’ thoughts was a challenge; this was mitigated by paraphrasing and clarification during interviews to ensure that responses were correctly understood.

## Conclusion

To our knowledge, this study is the first to explore cultural safety in ACP through the experiences of IENs in Ontario, Canada. IENs in this study acknowledged the significance of culture on the ACP process and sought to maintain cultural safety through various practical methods. Maintaining a stance of cultural humility, approaching ACP conversations in a cautious manner, and seeking to empower clients and families are some culturally safe ACP practices carried out by IENs in their practice. Facilitating nurses in their development of cultural humility practices and providing opportunities for ACP training is necessary. Clear guidelines and nursing standards should be developed to establish the role of nursing in the ACP process. Existing and future ACP materials and documents available to nurses and the general public must acknowledge the holistic nature of ACP and incorporate cultural safety. An overall need exists to better equip nurses in carrying out culturally safe ACP in their practice.

## Appendix A

Interview Questions

1. Where did you receive your nursing education?
2. How long have you worked in Ontario? Have you worked anywhere else other than Ontario (other parts of Canada or other countries)?
3. How long have you been a nurse (if participant has worked in various areas, ask how long in each place)-any other jobs in the healthcare field?
4. What type of setting do you work in (urban/rural; hospital (ask which floor/speciality), community (ask about specific type of community setting))
5. How many languages do you speak?
6. What is your understanding of what ACP is?
  ▪ How/where did you learn about ACP?
  ▪ Discussion about ACP, as well as clarifying, reaffirming, and explaining the concept of ACP with the participant will take place.
7. What was your experience engaging in ACP back home?
  ▪ Role of the participant in ACP discussions
  ▪ Other individuals involved in ACP discussions
  ▪ When is it normally initiated?
8. What was your experience engaging in ACP in your current work/role here in Ontario?
  ▪ Role of the participant in ACP discussions
  ▪ Other individuals involved in ACP discussions
  ▪ When is it normally initiated?
9. Are there any differences or similarities in how you engaged in ACP between these two experiences?
  ▪ Probe: What are these differences or similarities?
10. Think about the first time you engaged in ACP with persons/family in Ontario. What did that look like?
  ▪ Is that experience different from how you are approaching ACP engagement now? (If yes, what do you think brought about this difference in your practice?)
11. Can you tell me about the last time you engaged in ACP with persons/family in Ontario?
  ▪ Probe: What approaches did you take?
  ▪ Probe: What challenges did you anticipate or face?
12. In your opinion, does one's ethnocultural background influence their engagement in ACP discussions?
13. How did/does your own culture, values, and traditions influence(d) the way you do ACP with persons and families in your practice as a nurse?
14. Did your ACP experience look different with someone from your own cultural/linguistic background vs someone who wasn’t?
15. How did your formal and learned professional knowledge contribute to your ACP engagement with persons/family?
  ▪ Culturally competent training/ workshops
16. Prior to viewing the ACP workbook that was sent to you before the interview, have you ever heard of Speak up Canada or this workbook?
  ▪ What did you find was well laid out and/or beneficial in the workbook?
  ▪ Do you think this workbook is culturally inclusive?
    ▪ Probe: Does it meet cultural needs of individuals?
    ▪ Probe: Does it facilitate ACP that is culturally safe?
  ▪ What can be included in this booklet to ensure that this workbook is culturally inclusive and meets the needs of culturally diverse individuals during ACP?
17. Would you use this workbook to engage in ACP with persons/families?
18. Have you ever used any other ACP tools or workbooks when engaging in ACP with persons/families?
  ▪ If yes, what are these resources? How did you find them?
  ▪ If no, is there a reason why you do not use ACP tools?
19. What (cross) culturally safe ACP practices/approaches can help preserve and maintain persons/families’ cultural values and beliefs?
20. How can ACP engagement be structured/re-structured in order for nurses to engage in culturally safe ACP practices with persons/families?

Probe: What changes need to be made to current practice to better facilitate ACP with culturally diverse individuals?
